# Pre-school aged children are exposed to *Schistosoma* through Lake Kivu in Rwanda

**DOI:** 10.12688/aasopenres.12930.1

**Published:** 2019-02-22

**Authors:** Nadine Rujeni, Alex Mazimpaka, Musafiri Tumusiime, Elias Nyandwi, Gad Rutayisire, Pascal Kayiranga, Irenee Umulisa, Eugene Ruberanziza, Faith Osier, Francisca Mutapi

**Affiliations:** 1 Department of Biomedical Laboratory Sciences, University of Rwanda, Kigali, Rwanda; 2 Center for Geographic Information Systems , University of Rwanda, Kigali, Rwanda; 3 Rwanda Biomedical Centre, Kigali, Rwanda; 4 Department of Infectious Diseases, Universitätsklinikum Heidelberg, Heidelberg, Germany; 5 KEMRI-Wellcome Trust Research Programme/Centre for Geographic Medicine Research, Kilifi, Kenya; 6 Institute of Immunology & Infection Research, University of Edinburgh, Edinburgh, UK

**Keywords:** Schistosomiasis, exposure, pre-school children, snails, Lake Kivu, Rwanda

## Abstract

**Background:** Schistosomiasis is prevalent in many sub-Saharan African countries and transmission is through waters contaminated by infected snails. In Rwanda, although schistosomiasis is endemic, very few epidemiological studies exist; of these, schoolchildren have been the focus, neglecting pre-school-aged children (PSAC). Furthermore, malacological surveys to indicate the potential for transmission are scarce in the country. The aim of this study was to determine the prevalence of schistosomiasis among PSAC living on Nkombo Island in Lake Kivu and to map the distribution and infectivity of snails in the area.

**Methods:** Stool and urine samples were collected from children aged 1 to 4 years and tested for schistosomiasis using the Kato Katz and the point-of-care circulating cathodic antigen (POC-CCA) diagnostic techniques respectively. Snails were collected along the shores at five different locations with human-water contact activities and cercaria shedding was microscopically examined. GPS receivers were used to collect geographical coordinates and snail distribution maps were generated using ArcGIS. A questionnaire was used to assess water contact activities and frequency.

**Results:** A total of 278 PSAC were recruited. Overall, 9.5% (excluding traces) of the tested children reacted positively to the POC-CCA, although there were no ova detected in their stool via Kato Katz. The questionnaire revealed that 48.2% of parents/guardians use Lake Kivu’s water for household activities while 42.4% children are taken to the Lake shores daily. Overall, 13.5% of collected snails shed cercariae.

**Conclusions:** PSAC of Nkombo Island are exposed to *Schistosoma* parasites through contact with Lake Kivu, which hosts a number of snails shedding cercaria. Exposure is through recreational activities but also through bathing as safe water is scarce in the area. Health education of parents/guardians of these young children should be promoted and the national schistosomiasis control program should be integrated into water supply projects.

## Background

Schistosomiasis is a debilitating illness, due to *Schistosoma* parasites, ranking second amongst widespread parasitic diseases in sub-Saharan Africa ^1^. In the World, around 120 million people present schistosomiasis symptoms and an estimated 2.8 million years are lived with disability due to the disease ^2^. Ninety percent of the infected population live in sub-Saharan Africa, where *S. mansoni* and *S. haematobium* are the predominant species ^3^. The infection is acquired via infected stagnant waters that contain cercaria (schistosome larvae) shedding from snails, the intermediate hosts.

Schistosomiasis control programs have long been focusing on school children, overlooking younger pre-school-aged children (PSAC). However, in the last two decades, several studies have highlighted the important burden of the disease among PSAC in a number of countries in sub-Saharan Africa ^4– 8^. For instance, in the East African region, along the Ugandan shoreline of Lake Victoria, schistosomiasis prevalence of up to 86.0% among children under 3 years old have been reported ^9^. Another study reported a 62.3% prevalence for intestinal schistosomiasis in villages along the shores of Lakes Albert and Victoria in Uganda ^9^. In Kenya, a study reported a 14.0% prevalence among infants aged 1 year ^10^. Nevertheless, little to no urinary schistosomiasis was reported in a study conducted on Zanzibar Island in Tanzania among pre-school children subsequent to limited contact with infected water bodies ^11^.

In Rwanda, schistosomiasis has been reported several years ago, though a national control program was only established from 2008 ^12^. Nevertheless, data on the prevalence of schistosomiasis among PSAC in the country are scarce, despite the existence of an atlas showing the distribution of the infection in school children ^13^. Thus, the level of exposure and the national prevalence of schistosomiasis in PSAC are not documented and yet, their exposure patterns may differ from those of school children because of their differing behavior ^14^.

Exposure patterns to *Schistosoma* parasites are dependent on the availability and infectivity of snail intermediate hosts in each area. In Rwanda, although epidemiological studies ^15, 16^ have highlighted the presence of *Schistosoma mansoni* infection, the distribution and infectivity of snail intermediate hosts in most endemic areas are unknown.

Exposure patterns to *Schistosoma* parasites are topical because they may shape the host’s acquired immunity ^17– 19^, though the age also significantly influences the type and magnitude of the immune response ^20, 21^. Indeed, acquired immunity to schistosomiasis is skewed to a Th2 phenotype with the production of IgE responses ^19, 22^. However, the balance IgE/IgG4 seems to determine whether individuals become resistant or susceptible to infection/re-infection, and this balance is dependent on exposure levels and host age ^18, 19, 22^.

The current study aimed to determine the prevalence of schistosomiasis among PSAC living on Nkombo Island, located in the Eastern Province of Rwanda. The island is surrounded by the Lake Kivu and a previous study has reported a prevalence of 62.1% among schoolchildren ^16^, ranking the island at the top of schistosomiasis foci so far investigated in Rwanda. The second aim was to determine the level of exposure and the potential for transmission in the lake by assessing human water contact activities as well as snail distribution and infectivity.

## Methods

### Study area

**Figure 1.**
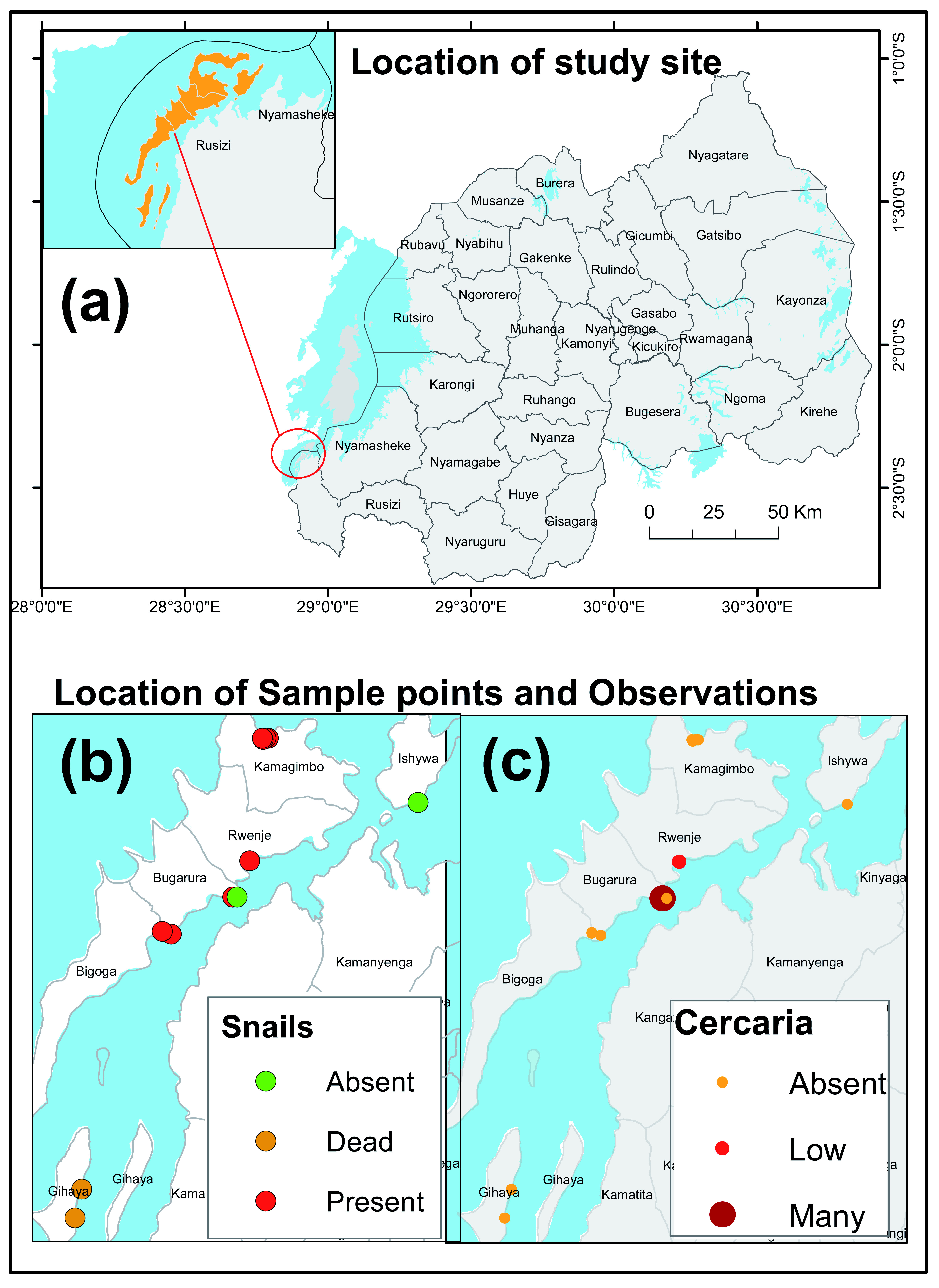
Geographical location maps of study areas ( **a**) and the distribution of snails and cercariae ( **b, c**). Geographical coordinates data were collected using GPS receivers and translated into maps using ArcGIS 10.4.

Nkombo Island, geographically located at 2° 23' 32" South and 28° 54' 35" East, is the largest island of Rwanda (21 km ^2^), situated in Lake Kivu in the Western Province, in Rusizi District ( Figure 1a). The Island hosts about 20,000 inhabitants with a meagre income, mainly coming from fishing. Like in many parts of the country, the area is highly endemic for soil transmitted helminths. The island is classified as a hyper endemic area for schistosomiasis based on a primary schools’ survey ^16^. The Island is subdivided into 6 Villages, namely Bigoga, Bugarura, Gihaya, Ishywa, Kamagimbo and Rwenje ( Figure 1b, c).

### Study aims

The main aim of this study was to determine the level and pattern of exposure to *Schistosoma* among pre-school aged children living on Nkombo, the largest Island of Lake Kivu.

Specific objectives were:

- To determine the prevalence of schistosomiasis using the Kato Katz and the POC-CCA diagnostic techniques
- To map snail distribution and infectivity along the lake shore
- To identify lake water contact activities and frequency through questionnaire and observations

### Study design and population

This was a cross-sectional study conducted in August 2016 on children aged 1 to 4 years who were permanent residents of Nkombo Island. Parents of pre-school aged children were sensitized about the study and were called at study sites by community health workers on a voluntary basis. A convenience sample of 278 children from different villages were included and provided stool samples. Of these, 211 also provided urine samples. For sample collection, five study sites were randomly pre-defined using the layers of lowest administrative units and spatial sampling tool of ArcGIS 10.4 software. The identified sample points were further adjusted with the help of local health workers for ease of accessibility. The number of children per residential village is shown in Table 1. A questionnaire ^23^, was administered to parents/guardians accompanying children at the study site to assess the type and frequency of water contact activities (for both parents and children).

**Table 1.** Age and gender distribution of the study population.

Age	Gender	N	CCA results, n
1 year	Female	20	13
	Male	19	11
	Total	39	24
2 years	Female	33	27
	Male	30	22
	Total	63	49
3 years	Female	40	27
	Male	48	43
	Total	89	70
4 years	Female	45	34
	Male	43	34
	Total	89	68
Total		278	211
Mean age		2.8	2.86
Sex ratio		1.01:1	1.09:1

### Ethical approval and consent to participate

Ethical permission was obtained from the Institutional Review Board of the College of Medicine and Health Sciences at the University of Rwanda, and the study was also approved by the Rwanda Biomedical Centre (RBC) of the Rwandan Ministry of Health. Parents/guardians of the study children signed a consent form before enrollment in the study and after a thorough explanation of the study aims and procedures was given. In agreement with the RBC, and because of the high level of geo-helminthiases in the area, all children were offered a single dose of Mebendazole after providing samples and those who were found to be positive for schistosomiasis were given Praziquantel through the Health Center. All participants were free to withdraw from the study at any time. 

### Parasitological examination

A single stool and urine sample was collected between 11 am and 2 pm, from each participating child, in plastic containers (distributed on the day of collection) and were processed on the day of collection. Kato Katz and the point-of-care circulating cathodic antigen (POC CCA, Pretoria, South Africa) diagnostic techniques were used (on stool and urine samples, respectively) to determine the prevalence of schistosomiasis ^24^. For urine samples, a drop was placed on a POC CCA cassette and results were read after 20 minutes as recommended by the manufacturer. For the Kato Katz, a single stool sample was collected, and three different slides were prepared (and mounted with cellophane sheets pre-soaked in malachite green solution) and read by three different laboratory technicians. Because of the remoteness of the island (and study sites) and the budget limitation, it was logistically impossible to collect more than one stool/urine specimen per participant. 

### Snail scooping and cercaria shedding

At the five study sites, water contact areas were identified (through independent observations) and two to three transects (approximately 10 m apart) per site at the shores were selected for snail sampling. Transects were thoroughly scooped, using cotton mesh scoops, by two trained field collectors.

Shedding cercaria was performed using an optimized protocol adapted from published studies ^25, 26^. Briefly, captured snails were individually kept in shedding pots containing lake water overnight, after which they were placed in new shedding pots containing distilled water. Shedding pots were placed under direct sunlight for 20 minutes, then in the dark for 15 minutes and into the light again. Using a microscope, the distilled water in the shedding pots was screened for cercaria. Non-shedding snails were kept in lake water (in shedding pots) overnight again and re-exposed to sunlight (alternatively with darkness) the following day.

### Data analysis

Statistical analysis was done using SPSS Statistics 21 (IBM). Binary variables were compared using Chi-square tests. Statistical tests with p-values ≤0.05 were considered significant. Geographical coordinates were captured using Garmin ground receiver GPS and further displayed as points map under ArcGIS 10.4 software environment ( Figure 1a, b).

## Results

### Prevalence, age and gender distribution of the study population

Children were aged between 1 and 4 years, with a mean age of 2.8 and sex ratio (male:female) of 1.01:1 ( Table 1). Based on the Kato Katz technique, no ova of *Schistosoma* were seen on prepared slides from the single stool that could be collected (though heavy infections with soil transmitted helminths were observed). However, the CCA diagnostic technique indicated a prevalence of 9.5% ( Table 2). There was no significant difference between male and female participants, nor between the different ages. Raw data for this study are available on Open Science Framework ^23^.

**Table 2.** Prevalence of schistosomiasis and distribution per age and gender.

Variable	CCA positive, n	Total, n	χ ^2^-value	p-value
**Age (years)**			0.387	0.943
1.0	3	24		
2.0	5	49		
3.0	6	70		
4.0	6	68		
**Total**	**20 (9.5%)**	**211**		
**Sex**			1.466	0.226
Male	13	97		
Female	7	94		

### Water contact activities and parents’ knowledge

The questionnaire administered to parents/guardians of study children indicated that the lake and a newly constructed borehole were the main sources of water for household activities and for bathing children ( Table 3). Overall, 38 (13.7%) parents reported bathing their children at the lake shore, while 118 (42.4%) parents reported that their children are taken to the lake shore every day ( Table 4). Notably, 210 out of the 260 (80.8%) interviewed parents/guardians said that they did not know schistosomiasis (Bilharzia).

**Table 3.** Water contact activities: bathing and household activities.

Location	Where children are bathed, n (%)	Fetching for household activities, n (%)	Fetching for bathing children, n (%)
Lake	38 (13.7%)	134 (48.2%)	134 (48.2%)
Borehole	0	142 (51.1%)	143 (51.4%)
Home	229 (82.4%)	0	0
Lake and borehole	0	2 (0.7%)	1 (0.4%)
Home and borehole	10 (3.6%)	0	0

**Table 4.** Water contact activities: frequency of lake water contact.

Frequency	N
Everyday	118
Never	77
Sometimes	83

There were differences between the levels of water contact in the different Villages. Indeed, the biggest number of parents reporting that their children are taken to the Lake was from the Villages Gihaya and Ishywa. In addition to bathing, young children also play in and around the lake water, accompanied by their elder siblings or parents.

### Snail distribution, infectivity and association with human infection

Collected snails were of *Biomphalaria* sp. and *Bulinus* sp. species based on published identification keys ^27^, but only *Biomphalaria* shedded cercaria. A total of 52 snails from 4 study sites were analyzed, and 7 of them (13.5%) shedded cercaria. At one study site, all snails were found dead and therefore could not shed. Snails collected near Bugarura and Rwenje Cells shedded cercaria while those collected from elsewhere did not shed cercaria ( Figure 1). Parasitological data indicated a slightly higher prevalence of schistosomiasis in children from these cells.

Since snails were collected from one side of the Island (with a poor representation of residential Cells, see Figure 1), children’s residential areas were categorized into three residential units according to the distribution and infectivity of surveyed live snails: Residential unit 1 being the area closest to where live snails were found and cercaria shedded; residential unit 2 the area closest to where there was no snail along the lake shore and residential unit 3 the area closest to where live snails were found but cercaria did not shed. In residential unit 1 (where snails shed) but not the other residential units, infection status was significantly associated with the frequency of lake water contact ( Table 5).

**Table 5.** Exposure frequency and infection status.

Residential Units	Schistosomiasis status	Frequency of lake contact			Total	χ²	p
		Never	Sometimes	Everyday			
Residential Unit 1	Uninfected	31	25	16	72	**9.07**	**0.011**
	Infected	5	0	7	12		
Residential Unit 2	Uninfected	6	12	29	47	0.731	0.694
	Infected	0	1	1	2		
Residential Unit 3	Uninfected	12	27	25	64	1.367	0.505
	Infected	0	3	3	6		

## Discussion and conclusions

With increasing evidence that pre-school aged children are affected by schistosomiasis, and that their exposure patterns may differ from those of school-aged and older children, it becomes important to assess the level of infection/exposure and associated morbidity in each endemic setting. Unfortunately, in Rwanda, to our knowledge, no single study has investigated the national schistosomiasis prevalence in pre-school aged children. The little number of existing published and non-published studies focused on school based surveys. Indeed, the national schistosomiasis control program focuses on school aged children (with some extrapolation to adult individuals), leaving out pre-school aged children.

The current study investigated the level of schistosomiasis among pre-school children living on Nkombo Island, an area that has been ranked first among schistosomiasis foci in Rwanda based on primary school surveys ^16^. Parasitological examinations using the Kato Katz revealed that no child was excreting *Schistosoma mansoni* eggs in their stool. However, using the POC-CCA diagnosis, 9.5% (excluding traces) of children tested positive. This finding is consistent with the fact that these children may carry pre-patent infections or light infections that are undetected by the less sensitive Kato Katz technique ^28^. The possibility for an increased low sensitivity of the Kato Katz technique by the single stool sample tested instead of the recommended three cannot be ruled out. The lower level of infection observed compared to infection levels reported in neighboring countries ^9, 10^ may be associated with the introduction of water pumps (borehole) that are used by nearly half of the population, as indicated by the parents/guardians of our study population. That is in line with the reported improvement of living conditions in Rwanda, as a result of significant policy achievements in the last two decades ^29, 30^. Nevertheless, the current study highlights spatial exposure dissimilarities along the lake shores, possibly linked to lower sanitation levels in some areas compared to others. The contribution of water physico-chemical parameters as well as other environmental factors in the distribution of snail intermediate hosts should be explored.

The malacological survey conducted indicated that snails of the genus *Biomphalaria* were widely distributed in the study area, though cercaria shedding was very focalized. It was reported by parents/guardians of the study children that the latter were often taken to the lake shores and that the lake water was highly used for household activities. As expected, the biggest number of infected children lived in residential units where live snails were shedding cercaria. Furthermore, the frequency of water contact was associated with infection status among children who lived in this residential area, consistent with published data ^31^.

It is important to note that *Schistosoma* parasites may still be absent in a large area of Kivu Lake as demonstrated by the absence of cercaria in samples and the low levels of human infection in most residential areas despite very frequent water contacts. This suggests that coupling MDA activities with a wider provision of clean water for bathing and household activities may shape the way to schistosomiasis elimination in and around the Lake. In addition, health education amongst parents/guardians on the Island, for instance the effects of hygiene and defecation behavior, would be highly beneficial as the majority did not know anything about schistosomiasis and its transmission route. It is worth noting that the population on this Island depends heavily on fishing and the lack of latrines near the work places may imply that open defecation is still frequent.

Overall, our findings indicate a significant infection with *Schistosoma mansoni* for pre-school aged children in Rwanda, although this is lower compared to some areas of neighboring countries. The study also indicates spatial dissimilarities in the distribution and infectivity of snail intermediate hosts, suggesting contribution of extrinsic factors such as physical, chemical and ecological aspects of their niche.

Among interviewed parents/guardians, 81% are ignorant about schistosomiasis transmission risks, further increasing the risk of child exposure to *Schistosoma* parasites. The national schistosomiasis control program should consider different age groups (including under five children) in the future schistosomiasis surveys and treatment strategies. Furthermore, the distribution of clean water for bathing and for household activities, combined with health education would shape the way to schistosomiasis elimination on the Island.

## Data availability

### Underlying data

Open Science Framework: Schistosomiasis in Rwanda. https://doi.org/10.17605/OSF.IO/ASQ3Z ^23^. Data are contained in file Dataset.Nkombo.sav.

### Extended data

Open Science Framework: Schistosomiasis in Rwanda. https://doi.org/10.17605/OSF.IO/ASQ3Z ^23^. The questionnaire is found in file: Questionnaire_Exposure MS.docx.

Data are available under the terms of the Creative Commons Zero "No rights reserved" data waiver (CC0 1.0 Public domain dedication).
