# Supplementary material for: Pre-school aged children are exposed to Schistosoma through Lake Kivu in Rwanda
Source: AAS Open Res. 2019 Feb 22;2:7. [Version 1] doi: 10.12688/aasopenres.12930.1 (PMC9648362; doi:10.12688/aasopenres.12930.1)
Supplement: Peer review history

# Peer review: Reviewer response for version 1

**DOI:** 10.21956/aasopenres.14006.r26780

**Published:** 2019-03-06

**Authors:** J. Russell Stothard

**Affiliations:** 1 Department of Parasitology, Liverpool School of Tropical Medicine, Liverpool, UK

This is a carefully written report arising from a targetted epidemiological survey, which is focused on revealing a new disease problem in Rwanda (i.e. intestinal schistosomiasis in children younger than school-age). It is a first report from this country and adds to the growing awareness that young children are infected yet do not have sufficient access to praziquantel treatment.

 Combining snail surveys is an important aspect which makes the overall interpretation much stronger. Especially as schistosome cercariae (note plural of cercaria) were seen. This gives greater insight into the urine-CCA results as being active infections although not yet egg patent by Kato-Katz examination. It is known that there is active transmission of intestinal schistosomiasis here in Lake Kivu and also other studies using urine antigen tests have revealed more infection/disease than previously thought (see Colley *et al.*, 2017 ^1^). We recently reported that the urine-CCA test was a solid tool for detection of infections in young children (see Sousa-Figueiredo *et al.*, 2013 ^2^). It might be worth elaborating on these points in the discussions.

 Foremost I was delighted to see that praziquantel treatment was provided to children who were CCA-positive. It shows that in principle a 'test and treat strategy' is possible without recourse to mass drug administration. I am concerned that the awareness of the disease was low and suggest the authors also think of ways to improve this as it will be central in future community mobilization and uptake of treatments offered.

Is the work clearly and accurately presented and does it cite the current literature?

Yes

If applicable, is the statistical analysis and its interpretation appropriate?

Yes

Are all the source data underlying the results available to ensure full reproducibility?

Yes

Is the study design appropriate and is the work technically sound?

Yes

Are the conclusions drawn adequately supported by the results?

Yes

Are sufficient details of methods and analysis provided to allow replication by others?

Yes

Reviewer Expertise:

Parasitology/epidemiology

I confirm that I have read this submission and believe that I have an appropriate level of expertise to confirm that it is of an acceptable scientific standard.

## References

[1] : Schistosomiasis is more prevalent than previously thought: what does it mean for public health goals, policies, strategies, guidelines and intervention programs?. *Infect Dis Poverty* .2017;6(1) : 10.1186/s40249-017-0275-5 63 10.1186/s40249-017-0275-5 28327187PMC5361841

[2] : The urine circulating cathodic antigen (CCA) dipstick: a valid substitute for microscopy for mapping and point-of-care diagnosis of intestinal schistosomiasis. *PLoS Negl Trop Dis* .2013;7(1) : 10.1371/journal.pntd.0002008 e2008 10.1371/journal.pntd.0002008 23359826PMC3554525

# Peer review: Reviewer response for version 1

**DOI:** 10.21956/aasopenres.14006.r26781

**Published:** 2019-03-05

**Authors:** Daniel G. Colley, Carl H. Campbell

**Affiliations:** 1 Center for Tropical and Emerging Global Diseases, Department of Microbiology, University of Georgia, Athens, GA, USA; 2 University of Georgia, Athens, GA, USA

Comments of Carl H. Campbell, Jr. and Daniel G. Colley:

 This manuscript reports on research done on Nkombo island in Lake Kivu, Rwanda to determine the prevalence of schistosomiasis in 1-4 year-old children and in snails collected along the shores of the island in areas frequented by local inhabitants. It also reports on water contact activities and parent’s knowledge of schistosomiasis. There have been other reports of schistosomiasis in the under 5 group, but none previously from Rwanda. Thus the authors are to be congratulated for conducting this research on this important and often neglected population. Prevalence data for this population is needed more broadly and is especially needed for countries that have began to control morbidity and are moving towards sustaining control and considering moving towards elimination. The background provided along with the appropriate citations highlights the need for Rwanda as well as other countries to better understand the disease prevalence of this pre-school age population, by inclusion of this population in future survey/mapping activities. The Study Aims and specific objectives are clear and very appropriate.

Comments, questions and recommendations:

  

1. The prevalence of schistosomiasis in the children is documented by the now WHO-endorsed Point-of-Care Circulating Cathodic Antigen (POC-CCA) assay, but the results are reported excluding trace reactions, contrary to recommendations by both WHO as well as the manufacturer. Upon examination of the source data (which could only be viewed by someone with SPSS software or after exportation from SPSS to another program) the authors only recorded 0, 1 and 3 readings of the POC-CCA assays. This means it is not feasible to compare these data with those of other studies using the POC-CCA assay in comparable populations and that often report the more standard 0, trace, 1+, 2+, 3+ readings.
  In the results, it is strongly recommended the authors include a column that depicts the total number of ‘positive’ POC-CCA results, including ‘Trace’ as positive. The reasons include **a)** the manufacturer recommends including Trace results as positive, and **b)** an analysis in school-age children in Burundi using latent class analysis (Clements *et al.*, 2018 ^1^) indicates that approximately 50% of Trace results are True positives. An analysis using ‘Trace as positive’ in addition to the current analyses as ‘Trace as negative’ would make a very important addition to the manuscript in regard to desired generalized nature of the data.
2. If possible, the Batch number(s) and expiration date(s) of the POC-CCA assays used should be included. The reason is that there was a period-of-time when the manufacturer was attempting to optimize the assay in order to reduce the percentage of Trace results. Unfortunately, some of these less sensitive Batches were provided and should not have been used for ‘mapping’ purposes. This study was done at a time when it could have been influenced by these relatively insensitive Batches, thus making the Batch numbers important.
3. In the brief description of the use of the POC-CCA, the methods say one drop of urine was tested. Did the investigators also add one drop of buffer, as indicated by the manufacturer for the original POC-CCA assay? If so, they need to add that they added the drop of buffer. The most recent POC-CCA assay uses two drops of urine and no buffer, so clarification would be helpful.
4. The authors’ main finding is reported as 9.5% prevalence in the target age group by POC-CCA in the face of 0% positive by Kato-Katz stool examinations. This situation has been previously reported from Kenya (Shane *et al.*, 2011 ^2^) with additional data showing that antibody levels (by SWAP ELISA) as well as CCA positivity precedes detectable egg excretion in 1-4 year-old children. This is an important finding that the authors have now nicely confirmed. This does not mean the current findings are not worthwhile, on the contrary it means that they are critical corroborating data from a different place, and that they should take credit for that and make that clear.
5. Based on Table 2 it can be calculated that the prevalence seen in children ages 1, 2, 3 and 4 are 12.5%, 10.2%, 8.6% and 8.8%, respectively. The authors should comment on why the prevalence does not increase with age, as seen in other studies.
6. It is unclear what the point of the paragraph about immune responses is in the context of the study presented. No immunologic data are provided, and thus the paragraph that begins “Exposure patterns to…” and references 17-22 seem irrelevant to the topic presented and the rest of the manuscript, and should be omitted.
7. The malacology findings are interesting, but also may, at some point, require molecular conformation of the *Schistosoma spp*. of shed cercariae. This is not a major point, but whenever possible, the co-existence of *S. rodhaini* or other forked-tail schistosome cercariae in areas with *S. mansoni* make the molecular species identification an important part of current studies, and this might be mentioned. The distinct distribution reported in terms of shedding and non-shedding snails may well be dependent more on where people defecate along the shore line, rather than where they bathe or swim and this might be mentioned if data were collected on this point.
8. What was the rationale to test 75% of the total enrolled population by POC-CCA versus the 100% by Kato-Katz?
9. Did the different levels of water contact, especially in the two villages (Gihaya and Ishywa) mentioned, also reflect higher prevalence among the PSAC?  A simple descriptive table might demonstrate that very well.
10. If the snail species for *Biomphalaria *and especially for *Bulinus* species is known, it would be useful to include.
11. In the discussion, it was very good that the authors addressed both the gaps in knowledge as well the need to for understanding the level of WASH in these communities.

 

Additional responses to AAS questions:

- The work is clearly presented, and for the most part appropriate citations are included. If the ‘Trace as positive’ analysis can be added, then additional references as noted (WHO; Clements *et al.*, 2018 ^1^) should be cited.
- The study design was excellent and appears to be technically sound.
- As noted above, the methods need some further explanation in regard to the POC-CCA assays and the Batch numbers to assist in comparisons by others.
- The interpretation of the data is appropriate.
- It appears that the authors have made the source data available in order to help others replicate this important work but it would perhaps make it more accessible to a wider audience if the SPSS data exported into Excel could also be made available to non-SPSS users/owners.
- The conclusions made are supported by the results. The additional analyses of including ‘Trace as positive’ would increase the usefulness of the results for others and strengthen the manuscript considerably.

 Based upon this review, our recommendation is **Approved.**

Is the work clearly and accurately presented and does it cite the current literature?

Partly

If applicable, is the statistical analysis and its interpretation appropriate?

Yes

Are all the source data underlying the results available to ensure full reproducibility?

Yes

Is the study design appropriate and is the work technically sound?

Yes

Are the conclusions drawn adequately supported by the results?

Yes

Are sufficient details of methods and analysis provided to allow replication by others?

Partly

Reviewer Expertise:

NA

We confirm that we have read this submission and believe that we have an appropriate level of expertise to confirm that it is of an acceptable scientific standard.

## References

[1] : Latent class analysis to evaluate performance of point-of-care CCA for low-intensity Schistosoma mansoni infections in Burundi. *Parasites & Vectors* .2018;11(1) : 10.1186/s13071-018-2700-4 10.1186/s13071-018-2700-4 29475457PMC5824563

[2] : Evaluation of urine CCA assays for detection of Schistosoma mansoni infection in Western Kenya. *PLoS Negl Trop Dis* .2011;5(1) : 10.1371/journal.pntd.0000951 e951 10.1371/journal.pntd.0000951 21283613PMC3026766
